# Round the Hospitals

**Published:** 1920-02-07

**Authors:** 


### ROUND THE HOSPITALS.

Two appeals have been made in the course of the last week for objects near to the heart of all nurses. The Edith Cavell Homes of Rest Council announce that through the generosity of Mr. W. G. Groves they have opened a new Home for nurses at Windermere, capable of providing one month's rest in the year to 120 nurses, giving each a separate bedroom. It will be specially valuable in providing for the rest of north country and Scottish nurses. To endow it the sum of £20,000 is required. A corresponding effort is being made to secure a convalescent home for the use of V.A.D. members, of whom there are still some 4,000 serving under the military authorities. The lease of "Hartsleap," where the V.A.D. convalescent home has been situated, has now run out, and Sir Arthur

**ROUND THE HOSPITALS**—(*continued*).

Stanley has appealed through the *Spectator*, a paper which has always warmly espoused the cause of the V.A.D., for a furnished country house with some twenty bedrooms and pleasant grounds to replace it.

Every nurse in the Kingdom should make an effort to secure a copy of the *Daily Telegraph* day by day and watch what the world is thinking and saying of the members of her profession. Financial reasons apart, nurses are deeply indebted to Lord Burnham for the searchlight he is throwing over nurses' deeds, prospects, and ideals. The Fund, which is already overtopping its first 100,000 shillings, is eliciting warm response from every section of the public, and it is impossible to read dry-eyed the evidence it affords of our nurses' heroism. Mrs. Martin Harvey's graphic sketch of the conditions which prevailed on the Western Front appeared on January 30, and should not be missed by any. The story of how the gassed men were nursed in the summer of 1918 was told on February 2 by Mr. Eric Howard, late sergeant in the R.A.M.C., and now attached to Mr. Martin Harvey's company at Covent Garden. It is one of the most poignant incidents in the whole campaign, and is indispensable to the future history of war nursing. Sir Henry Burdett, K.C.B., K.C.V.O., contributed on January 29 a record of observed facts relating to the nursing profession which reveals the fact that out of an average of some 2,000 nurses suffering from illness or overfatigue, one in every four is relatively young, and should be in robust health.

The soldiers are coming forward eagerly with their tribute to the nurses. One who has been "A Patient in Ten Hospitals" expresses his admiration and gratitude for "those magnificent and devoted women our war nurses," and "Ex-officer" had the same experience. A "poor man from the Rowton House" sends a shilling thanking God "for creating such noble women." In schools, factories, public departments, private houses, shops, and institutions of all kinds, shilling collections are being eagerly responded to. The printers' chapel of the *Daily Telegraph* has sent 100 shillings. Nurses are among the most enthusiastic to bear testimony to the needs and selfless devotion of their sisters in want. Dame Sarah Swift, matron-in-chief expresses her unqualified gratification and that of the whole nursing profession at the efforts made by the *Daily Telegraph* on behalf of suffering nurses. It is proposed that a day shall be set apart, February 16 is suggested, for publication in the *Daily Telegraph* of a list of contributions from matrons, sisters, and nurses. Many grateful patients offer tribute in money and gratitude. Numerous shipping firms have opened collections, and all ships in commission, as well as all regiments in the United Kingdom, have been furnished with collecting sheets, as have also a thousand branches of the London City and Midland Bank. A grand carnival is to be given by the National Sporting Club at Holland Park Skating Rink on Wednesday, February 18, in aid of the fund. Provincial papers are taking up the cause, and there is ample evidence that the country is rousing on behalf of the nurses.

The letter from "Justice" in our last issue on the "Nurses' Eight-hour Week," is of a kind that does good. It is a page of real life. The writer exaggerates nothing, has no bitterness, is possessed by a burning zeal to save other women from the trials she has herself endured. How rare this attitude is in those who strive for reform none can tell but those who have invoked help in the struggle to get things righted. Over and over again the men and women who work for the amelioration of the nurse's lot encounter the opposite spirit; a tendency to embroider the evidence, vitiating all its value; an unbalanced judgment attributing evil motives to their fellow-sufferers; finally, and most hopeless, the attitude which may be defined as "*I* had to do it, why not they?" Personally, we have read no more moving plea for an eight-hour day for nurses than this quiet, simple letter.

We were quite sure that Lord Burnham's admirable appeal to the public on behalf of disabled nurses and the development of their profession would not pass without opposition from the short-sighted opponents of the College. The contention that "no other profession would tolerate" an appeal to public charity on behalf of its members is a little ill-timed at a moment when all the Bishops are making moving appeals for the rescue of many of the clergy from a condition of grinding poverty. "Self-respecting nurses," by whom we presume are meant members of the profession in the enjoyment of a fair salary, whose health is good and whose future is secured by assurance in the Royal National Pension Fund for Nurses, could not by any effort of self-sacrifice meet the calls on their purses for aid to their less fortunate sisters. There are three courses open to them. They may hide their heads in the sand and deny the existence of notorious distress. They may admit the facts and, denying any responsibility for their fellows, obstruct those who are trying to succour nurses in distress. Or they may throw themselves whole-heartedly into the work of inviting a wealthy and deeply sympathetic public to discharge their admitted obligations towards the women who have stood between them and the pains of death. Every nurse in the kingdom should make up her mind which of these three courses is the most honourable.

Lady Evelyn Cotterell presided over an interesting meeting of the Hereford Red Cross Branch on January 21, when several excellent schemes were approved. One of the Hereford Women's Detachments has decided to go into camp at the seaside in the summer for training, and a small grant was made for the hire of tents, the members being responsible for their own maintenance. The detachment, of course, includes trained

**ROUND THE HOSPITALS**—(*continued*).

nurses, and it is proposed that the morning shall be devoted to drill and instruction, leaving the rest of the day free. The proposal is eminently practical, and is likely to be imitated in many urban districts. We note also that the Branch is distributing its surplus stretchers, numbering about fifty, to outlying villages for use in cases of emergency. It is undoubtedly true that to have a stretcher at hand may obviate much suffering and injury, but it should be seen to that in villages thus provided regular instruction in ambulance work is available. Lifting stretchers in the right way does not come by nature.

A revised scale of salaries has been issued by the Durham County Council for the nursing staffs of their tuberculosis hospitals. It runs as follows, and is a considerable improvement on the former scale:—

| Status. | Commencing Salary. £ | Annual Increment. £ | Maximum Salary. £ |
|---|---|---|---|
| Probationers ... ... ... | 28 | 4 | 32 |
| Partially Trained Nurses ... | 35 | 4 | 47 |
| Trained Staff Nurses ... ... | 50 | 5 | 60 |
| Trained Staff Sisters ... ... | 65 | 5 | 75 |
| Matron (under 50 beds) ... ... | 80 | 10 | 100 |
| Matron (over 50 beds) ... ... | 150 | — | — |

We trust it will have the desired effect of bringing the staff up to full strength in these establishments. Durham is still very short of midwives. Unqualified women still practise under cover of so-called "emergency," and at least twenty trained midwives are needed to provide an adequate service.

Edith Cavell's hospital and training-school for nurses in Brussels, now known as the Ecole Edith Cavell, has been recently enlarged by a new ward containing twelve beds, soon to be increased to twenty-five. It was the gift of an American lady, Miss Heynemann, who worked devotedly among the Belgian soldiers in London during the war. It has been lately opened under the name of the California Ward, and an influential company gathered at the little ceremony, including the British and American ambassadors with their wives, the Belgian Minister for Foreign Affairs, and Dr. Depage, the original founder of the now famous school.

The East Lancashire Centre of the College of Nursing is rapidly advancing towards the completion of its weighty task, which included the raising of £10,000 towards the endowment fund of the College, and another £10,000 to form a fund for East Lancashire nurses. Already the first £10,000 has been handed over to the endowment fund, and this is in itself a notable triumph for Manchester and the district. Of the remainder, £4,000 is in hand, and none can doubt that, with the excellent organisation this centre possesses, the rest will be speedily forthcoming. The aim is to found a residential club in Manchester for the use of all East Lancashire nurses, with special terms for members of the College. Scholarships of £150 to £180 will be offered to train health visitors and sister tutors, and temporary and permanent help will be available for nurses in distress. It is a great help in this enterprise that the Lord Mayor of Manchester has accepted the chairmanship of the East Lancashire Centre, and that the Lady Mayoress, who is a fully-trained nurse, has been elected on the council. Everyone knows how much the Centre owes in initiative and driving force to Miss Sparshott.

The question of overtime for nurses in connection with a forty-eight-hour week opens up a rather difficult question. By the terms of the "Hours of Employment Bill," which was introduced last session, workers are to be entitled to a minimum payment known as "time and a-quarter" for all over-work; say 1s. 3d. an hour instead of 1s. But this provision is made on the assumption that workers are not lodged and boarded. The probationer's salary may be reckoned as £20 a year, which would make her average rate of payment per hour, allowing for a forty-eight-hour week with a month's holiday in the year, work out at about 2d. an hour. But with emoluments her salary amounts to at least £80 a year, which gives an average of 8d. an hour. At what rate will her overtime be reckoned? It will certainly be a very good thing for every one concerned if institutions are compelled to reckon out the exact value of the maintenance, uniform, and other emoluments supplied to their employees.

We are very glad to hear that the Stores and Transport Department of the British Red Cross have sent out notices to the county organisations which fed their supplies during the war asking them to organise working parties once more and register under the Red Cross. Lady Gosford, appealing to the county branches to resume work, says: "Children's hospitals, child-welfare centres, homes for those suffering from tuberculosis, all institutions for the sick and suffering, are in need of the assistance our working parties and home workers can render." The central workrooms, in Whitfield Street, W., will keep the county headquarters informed of the needs as they arise, and can supply wool and flannel at little over cost price. The appeal has met with a cordial response in the North Riding branch of the Red Cross, which is at once setting itself to its old tasks.

A highly interesting letter from a Cornish nursing sister now working in a Serbian village dispensary is given in the *Western Morning News* of the 25th ultimo. The writer was sent direct from England to the hospital at Nish, the headquarters of Serbian relief, where she had an eye ward and came in for a very gay time. Thence she was sent to a deplorable little orphanage in the same town for orphans and deserted children with Bulgar fathers, where she found the fifty-two children unhealthy and undisciplined, and helped the new matron to reorganise the establishment and deal with a measles epidemic. Then followed a two days' journey by cart up country to a little

**ROUND THE HOSPITALS—(*continued*).**

mountain dispensary, where a solitary nursing sister was holding the fort. Here we find a varied and strenuous work proceeding; beds for two in-patients and a ceaseless stream of out-patients pouring in from 6.30 A.M. till 10 P.M., while one of the two devoted women visits cases unable to be brought to the dispensary. The writer has nothing but kindness and generosity to record among her patients, and such a readiness to be healed that the simplest remedies banish the disease.

---

*Truth* has espoused the cause of the Queen Alexandra naval nurses, and calls attention to what it calls the " merciless sweating " in the Service. It is now a long time since a promise was made to redress grievances, raise the salaries, and shorten the hours, but the authorities move slowly. " These ladies still begin at £40, with a maximum prospect of £65 a year. Their messing allowance is 2s. 3d. a day. . . . They work eighty-four hours a week when on night duty, and sixty hours on day duty." The case for reform is very strong, and the fact that the Service is a small one, and that there is much competition to enter it, cannot excuse the delay in improving its conditions. In particular, the maintenance of twelve hours night duty is utterly inexcusable.

---

If the League of Red Cross Societies is to fulfil the hopes which have centred round it, it must do more than use its influence. We look to a time when it will have at its disposition in the various countries affiliated to it fully-organised units, with equipment ready, and a list of doctors, nurses, and other trained assistants from whom volunteers can be summoned to start off when wanted with the least possible delay. The American Red Cross Society has done nobly, but the workers need support and reinforcement from other countries, which do not appear to have been forthcoming. Meantime, the situation in Poland and Central Europe generally is that the population is dying in thousands without succour, and that the Central Red Cross organisation has (must we not own it?) disappointed expectation.

---

A statement appeared in the *Times* of January 20 from a correspondent in Warsaw, blaming the International Red Cross Society for inactivity in connection with the epidemic of typhus now raging in Central Europe. The reply, contributed recently by the Director-General of the League of Red Cross Societies, does not appear absolutely conclusive. The test of the International organisation will always lie, not in the list of influential persons attracted to its conferences, but in its ability to stimulate immediate succour in equipment and *personnel* when necessity arises. We cannot learn that any adequate attempt has been made to meet the urgent requirements of the populations among whom typhus and other fevers are taking deadly toll. But in justice to the League we would urge that it is as yet far too early to expect from this new central organisation the powers necessary to cope with this awful situation. The despatch of isolated parties of nurses or doctors would have been criminal folly. The despatch of a commission to investigate matters at close quarters, unless followed up by substantial aid, was certain to raise hopes impossible to realise, and this is exactly what has happened. The League has issued appeals for help to the Red Cross Societies of America, Australia, Belgium, Great Britain, Italy, Portugal, Rumania and Spain. It confesses itself impotent to do more than " use its influence."

---

The matron at Woolwich Infirmary (Mrs. Manning) has reported to her visiting committee that at the lowest estimate nine extra nurses would be needed to do the work under fair conditions, as the wards are at present, with the 48-hour week. She is carrying on now without relief or holiday-nurses. She is strongly opposed to the proposal to allow some of the nurses to live outside the institution, feeling convinced that it would be very inconvenient and lead to dissatisfaction and jealousies. The matron recommends that space should be gained by removing some of the chronic and senile cases to the workhouse wards, as suggested by Dr. Boulter some years ago. The number of these cases is larger than it should be in a training-school for nurses. We trust the Guardians may see their way to follow this sound advice. They have appointed a special committee to deal with it.

---

In approving the plans of the Southwark Guardians for the erection of a new wing to the Nurses' Home in connection with the infirmary, the Ministry of Health adds the proviso that at least half the number of bedrooms should be fitted with fire-places. This is a point to which we have always attached great importance. Not only is the fireplace a valuable ventilation-shaft, but it enables the room to be used by the occupant in cases of light illness or over-fatigue requiring confinement to bed. No central system of heating is so entirely reliable as not to break down when most wanted, and unless a good proportion of the bedrooms have fire-places considerable inconvenience and even suffering may result.

---

It has been decided to dissolve the Midland Matrons' Association, which was founded in 1910, and to form a Midland branch of the Association of Hospital Matrons. Miss Carless, of 87 Cornwall Street, Birmingham, has been appointed the honorary secretary of this branch, and all communications from intending members or others should be addressed to her. The subscription will be 2s. 6d. yearly, payable in January. The Midland Matrons' Association has closed with a credit balance of £7 16s. 8d., which has been transferred to the funds of the new branch. The final meeting of the former association was held on January 24 at the General Hospital, Birmingham.

